# Cardiac bronchus with a well-formed accessory cardiac lobe-a supernumerary lung?

**DOI:** 10.36416/1806-3756/e20260158

**Published:** 2026-09-14

**Authors:** Ester Labrunie, Gláucia Zanetti, Edson Marchiori

**Affiliations:** 1. Universidade Federal do Rio de Janeiro, Rio de Janeiro, Brasil.

A 34-year-old woman presented with a 5-day history of cough and fever. Chest CT showed normal lung parenchyma and revealed an accessory cardiac bronchus (ACB) containing rudimentary parenchymal tissue in its distal portion (Figure 1). 

**Figure 1:**
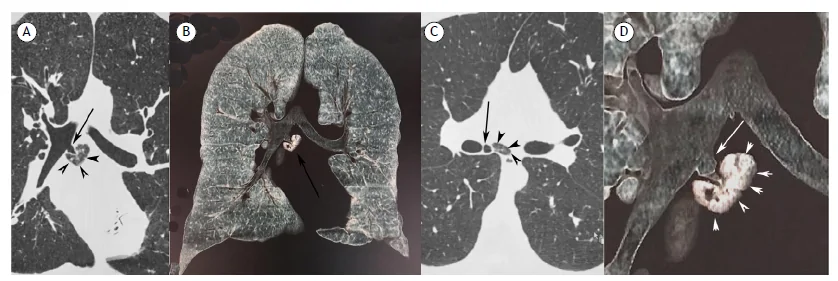
Coronal (A) and axial (B) CT reconstructions showing an accessory cardiac bronchus arising from the left main bronchus (arrows), associated with rudimentary parenchymal tissue (arrowheads). Three-dimensional coronal reconstruction (C) and a detail view (D) better demonstrate the relationships among the local structures.

Bronchial anomalies originating from the trachea or main bronchi are uncommon and are usually identified as incidental findings. Among these anomalies, the most frequently described are the accessory cardiac bronchus (ACB) and the tracheal bronchus. The ACB typically originates from the medial aspect of the right main bronchus or the bronchus intermedius and extends toward the heart. In most cases, it presents as a blind-ending structure. Nevertheless, in a minority of patients, the ACB may branch into small bronchiolar structures that terminate in cystic changes or are associated with underdeveloped pulmonary parenchyma, as observed in the present case.^1^ ^-^ ^3^

The majority of individuals with an ACB remain asymptomatic, and the anomaly is often detected during imaging examinations or bronchoscopic procedures performed for unrelated reasons. Clinical manifestations may occur when complications arise, including recurrent respiratory infections, empyema, hemoptysis, or, more rarely, malignant transformation. Diagnosis is generally established through fiberoptic bronchoscopy or CT. 

An important differential diagnosis is bronchial diverticulum or fistula, from which the ACB can be distinguished by the presence of normal bronchial epithelium and cartilaginous structures within its wall. No therapeutic intervention is necessary in asymptomatic cases discovered incidentally. However, surgical resection is recommended when the ACB is associated with recurrent infections or hemoptysis.^1^ ^-^ ^3^

In summary, familiarity with both normal bronchial anatomy and congenital bronchial variations is essential for pulmonologists and radiologists to ensure accurate diagnosis. Although the ACB is not inherently pathological, it may occasionally give rise to clinically relevant symptoms and complications.

## References

[1] Kuhtić I, Marušić A, Krešić E, Mandić T, Coce N, Butorac Petanjek B (2023). Accessory cardiac bronchus with associated lung parenchyma: rare congenital tracheobronchial anomaly. Acta Biomed.

[2] Gupta P, Prakash M, Aggarwal AN, Khandelwal N (2015). A rare bronchial anomaly presenting as a paracardiac mass. Lung India.

[3] Zanetti G, Hochhegger B, Guimarães MD, Marchiori E (2014). Accessory cardiac bronchus causing recurrent pulmonary infection. J Bras Pneumol.

